# Developing a Real Time Sensing System to Monitor Bacteria in Wound Dressings

**DOI:** 10.3390/bios2020171

**Published:** 2012-05-09

**Authors:** Malcolm J. Farrow, Iain S. Hunter, Patricia Connolly

**Affiliations:** 1 Department of Bioengineering, University of Strathclyde, Wolfson Centre, Glasgow, G4 0NW, UK; E-Mail: malcolm.farrow@strath.ac.uk; 2 Strathclyde Institute of Pharmacy and Biomedical Sciences, University of Strathclyde, Glasgow, G1 1XW, UK; E-Mail: I.S.Hunter@strath.ac.uk

**Keywords:** electrical impedance, infection, sensor, *Staphylococcus aureus*, wound

## Abstract

Infection control is a key aspect of wound management strategies. Infection results in chemical imbalances and inflammation in the wound and may lead to prolonged healing times and degradation of the wound surface. Frequent changing of wound dressings may result in damage to healing tissues and an increased risk of infection. This paper presents the first results from a monitoring system that is being developed to detect presence and growth of bacteria in real time. It is based on impedance sensors that could be placed at the wound-dressing interface and potentially monitor bacterial growth in real time. As wounds can produce large volumes of exudate, the initial system reported here was developed to test for the presence of bacteria in suspension. Impedance was measured using disposable silver-silver chloride electrodes. The bacteria *Staphylococcus aureus* were chosen for the study as a species commonly isolated from wounds. The growth of bacteria was confirmed by plate counting methods and the impedance data were analysed for discernible differences in the impedance profiles to distinguish the absence and/or presence of bacteria. The main findings were that the impedance profiles obtained by silver-silver chloride sensors in bacterial suspensions could detect the presence of high cell densities. However, the presence of the silver-silver chloride electrodes tended to inhibit the growth of bacteria. These results indicate that there is potential to create a real time infection monitor for wounds based upon impedance sensing.

## 1. Introduction

Wounds in the skin provide an ideal environment for bacteria to inhabit and colonise the wound [1]. This may lead to an established infection where the human immune system struggles to reduce the numbers of bacteria. Suspected wound infections require sampling of tissue and fluid, which is an invasive procedure in that it requires disturbing the dressing to swab the areas and exudate suspected of harbouring infection. Infection diagnostic systems used in hospital laboratories comprise mainly culture methods [2] and these are slow, costly and labour intensive [3]. This may delay the selection of an appropriate wound healing regime and increase the time before focused treatment can be started. Wound management would be greatly advanced if the presence of infection could be monitored in real time without disturbing the dressing.

Frequency of dressing changes varies from country to country, in some community settings dressings are changed daily but advanced wound dressings should only require changing every three to seven days unless wound exudate is particularly heavy. Thus there is opportunity for infection monitoring systems that do not require removal of the dressing. 

The electrical impedance of tissues has been studied in a range of applications which include excised tissues, diagnosis of cancer, nutritional assessment, and muscle and lung functionality [4,5]. During the 1970s and 1980s bacterial growth monitored by electrical impedance methods was studied extensively due to the prospect of automation [6,7,8,9,10,11]. More recently, research has concentrated on micro and nano devices to create possible point of care diagnostic devices [12]. However these would still require dressing changes or sampling of wound exudate. Studies that have combined electrical impedance and sensors, and that can be used without removing the dressing include a system to monitor the size of chronic wounds [13], and a product to monitor wound moisture levels [14,15]. 

Electrical impedance can be represented in the form of its magnitude (|*Z*|) and phase angle (*θ*), or in complex notation with resistance (*Z*') and reactance (*Z*"). These are related by Equations (1) and (2).


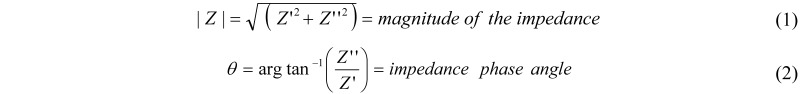


To examine the relative changes from the initial readings over time, the impedance measurements can be normalised. The normalised impedance modulus (|*Z*|*_n_*) at each frequency point is calculated by dividing the hourly reading (|*Z*|*_hour_*) by the reading at zero hours (|*Z*|*_time_* = 0), as shown in Equation (3). 


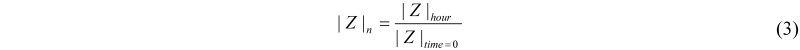


Thus |*Z*|*_n_* = 1 for all frequencies at time zero and deviations from this with time can be seen clearly on a graphical plot of |*Z*|*_n_* against frequency. Similarly the normalised phase angle (Equation (4)), resistance and reactance can be calculated.


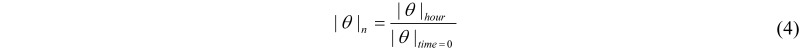


The normalised impedance data are analysed by comparing the normalised impedance profile plots (either normalised impedance modulus, or normalised phase angle, or both) against frequency in order to look for discernible differences caused by the presence of cells that were not present on the control plots. One feature of particular interest that emerges is the frequency band or location of any peaks in such graphs that occurred in the normalised impedance profiles over frequency. Normalised impedance data shows that different cell types demonstrate different characteristic responses from day to day in the growth cycle, that were consistent from culture to culture, with different locations of peaks in *Z_n_* with respect to frequency. Such consistent characteristic responses meant that cells could be typed in culture according to their normalised impedance profile across the frequency spectrum and that this might be extended to bacteria [16]. Impedance normalisation has also been used to monitor cell mobility, adhesion and alterations [17,18,19,20].

The aim of the current study was to investigate the feasibility of creating an infection monitor for wounds by combining the normalised impedance profile method with the moisture sensors previously described by McColl *et al*. [15]. The hypothesis was that these might have the potential to provide unique signature traces to indicate the presence of bacteria and differentiate species of bacteria. Wounds can produce significant quantities of fluid, with a moderately exuding wound producing 0.5 mL per square centimetre in a 24 h period [21]. This provides a suitable environment for bacteria to grow in the wound fluid and creates problems with exudate management in wound dressings. In addition the bacteria can attach and create biofilms on tissue components within the wound [22,23]. Therefore it is important to study bacterial suspensions and formation of biofilms on the electrode surfaces as part of the representation of possible scenarios in wounds. This paper describes the impedance profiles of bacterial growth in suspensions using screen-printed electrodes and several strains of *Staphylococcus aureus*, a species commonly isolated from wounds [3]. The results of the biofilm study will be detailed in a future companion paper.

## 2. Materials and Methods

The key experiments performed were to:

- Investigate the effects on impedance profiles against frequency with the sensor system for: *Staphylococcus aureus* growth in suspension, both in a standard culture broth, and when boosted by the addition of glucose to the same broth.
- Investigate the effects of: (a) lower inoculation density on the impedance profiles and (b) the effect of glucose concentration on the impedance profiles. These experiments were performed to explore the detection limits and understand the differences caused by the presence of glucose seen in the initial experiments.
- Test the changes in growth inhibition that might have been caused by the presence of the silver-silver chloride (Ag-AgCl) sensors by prewashing of the sensors to remove early release of silver chloride deposits
- Investigate the effect of different cell inoculation densities on the bacterial growth of three strains of *Staphylococcus aureus* in the presence of Ag-AgCl sensors.
- Investigate the effect of the application of voltage during the impedance measurements on the bacterial growth of three strains of *Staphylococcus aureus*. This was to further investigate the inhibiting effect seen in previous experiments, as previous results indicated that by performing the impedance measurements growth inhibition was increased.

The conditions and methods for each of the experiments are given below.

### 2.1. Bacteria

The bacteria used in the experiments were *Staphylococcus aureus*: one laboratory strain RN4220, and two clinical isolates SA081, and SA082 donated by the Robertson Trust Laboratory for Electronic Sterilisation Technologies, University of Strathclyde. All three strains were stored in 80% (v/v) glycerol at −80 °C. For use, the strains were streaked onto Mueller-Hinton agar (38 g/L) (Oxoid, Cambridge, UK) and incubated for 24 h before experiments. A single colony was grown in Mueller-Hinton broth (MHB) for 24 h and serially diluted to provide the inoculation density for each of the suspension experiments. 

### 2.2. Sensors

**Figure 1:**
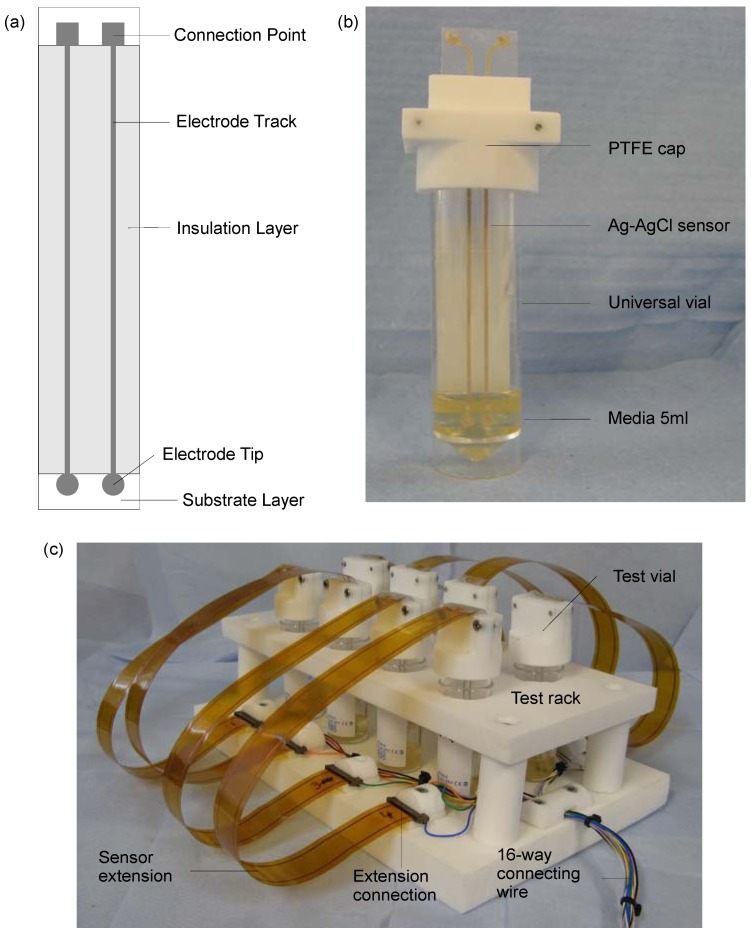
(**a**) The basic design of the screen-printed sensors. (**b**) The bacterial suspension test vial comprised a 30 mL universal vial, 5 mL of media, a specifically designed polytetrafluoroethylene (PTFE) cap and a Ag-AgCl sensor. (**c**) The test rig to allow parallel measurement of up to eight bacterial suspension test vials.

The disposable silver-silver chloride (Ag-AgCl) sensors were identical to those created for a clinical trial monitoring moisture levels underneath wound dressings [15] and made available for the project by Ohmedics Ltd. (www.ohmedics.com). The sensors were manufactured by screen printing a silver chloride ink onto a flexible polyethylene substrate. Each sensor had two planar electrodes with 3 mm diameter tips, 10 cm track lengths and connection pads (Figure 1(a)). The tracks were insulated with an adhesive layer of the polyethylene. Before the experiments, the sensors were sterilised in 70% (v/v) ethanol for 10 min and rinsed three times in sterile water [24,25].

### 2.3. Key Experiments

#### 2.3.1. Growth of Strain RN4220

The test rig consisted of one impedance culture vial with a sensor inside and one control culture vial with no sensor. The impedance vial consisted of a 20 mL universal vial (Greiner Bio-One, Stonehouse, UK) and a specially designed PTFE cap which held the screen-printed Ag-AgCl sensor suspended in the centre of the vial (Figure 1(b)). The control vial was a standard 20 mL universal vial. Both vials were filled with 5 mL of MHB. Both vials were held with a clamp stand on an orbital shaker 240 rpm (IKA, Staufen, Germany), inside an incubator, 37 °C (LEEC, Nottingham, UK). The sensor was connected via crocodile clips and BNC cables to a Solartron 1260 frequency response analyser (Solartron, Hampshire, UK). Impedance measurements involved frequency sweeps from 1 MHz to 0.1 Hz at an RMS voltage of 200 mV. 

Two initial impedance measurements were performed on the impedance vial before bacterial inoculation to read the background impedance, and then measurements performed once every hour for 17 h. Both vials were inoculated with a single colony of RN4220, approximately 1 × 10^6^ CFU∙mL^−1^, after the background impedance readings were taken. The final numbers of bacteria were calculated by colony counts. For the control vial no inoculation was performed. The control vial impedance readings were taken in order to record any conditioning effects of the MHB on the electrode impedance, and to observe such changes when no bacteria were present. The complete protocol was repeated over five independent experiments on this strain.

To examine whether the culture media affected the impedance measurements, the RN4220 growth procedure described above was repeated with the MHB media but with added glucose. In this case each vial contained 4.9 mL of MHB and 0.1 mL of 10% glucose (w/v) to create a 0.2% (v/v) glucose concentration. Five replicate experiments were also performed.

#### 2.3.2. Lower Inoculation Densities, Glucose and Pre-washing

To allow hourly impedance measurements to be performed on up to eight bacterial suspensions in parallel, a modified test rig was created (Figure 1(c)). The test vials were held in a specially designed PTFE rack on the orbital shaker. The sensors were connected to the 1260 frequency analyser via a Solatron 1281 multiplexor (Solartron, Hampshire, UK). Test vials were filled with 4.9 mL of media and inoculated with 100 μL of RN4220 culture containing approximately 1 × 10^3^ CFU∙mL^−1^, diluted from a 24 h MHB culture. Control vials containing bacteria and no sensors were used in parallel for each of the parameter variations.

The first parallel suspension experiment investigated: (a) the effect of lower inoculation density on the impedance profiles; (b) the effect of glucose concentration on the impedance profiles; and (c) whether pre-washing the sensors improved bacterial growth. The pre-washed sensors were washed for 24 h in MHB with the media refreshed three times prior to the start of the impedance measurements. The eight vials were: (1) MHB; (2) MHB, RN4220; (3) MHB-0.2% glucose; (4) MHB-0.1% glucose, RN4220; (5) MHB-0.2% glucose, RN4220; (6) MHB-0.4% glucose, RN4220; (7) MHB, with pre-washed sensor; (8) MHB, with pre-washed sensor, RN4220. Hourly impedance measurements were performed on all eight vials for 16 hours and the complete protocol repeated three times.

#### 2.3.3. Presence of Ag-AgCl Sensors

To investigate bacterial growth simply in the presence of silver from the Ag-AgCl sensors (without any application of voltage for impedance measurements) the bacteria were grown from four different starting cell densities for 24 h in systems in which the sensors were present but no impedance measurements were made. Two 20mL universal vials were filled with 4.9 mL of (MHB) to create a test and control vial. A sensor was placed in the test vial and both vials inoculated with 100 μL of media containing approximately 1 × 10^5^ CFU∙mL^−1^, diluted from a 24 h broth culture. An additional three pairs of test and control vials (no sensors present) were also tested with starting cell densities of 1 × 10^4^, 1 × 10^3^ and 1 × 10^2^ CFU∙mL^−1^. Cultures were grown at 37 °C for 24 h on an orbital shaker (240 rpm). The final numbers of bacteria were calculated using colony counts. The complete procedure was replicated three times for each of the strains RN4220, SA081 and SA082.

#### 2.3.4. Presence of Impedance Measurements

The second parallel suspension experiment investigated the influence of the application of voltage during the hourly impedance measurements on bacterial growth. Eight test vials were paired into four sets. All had sensors placed inside. Each pair of vials was inoculated from separate 24 h cultures and impedance measurements were only performed on one vial from each pair every hour for 24 h. This was repeated for each strain of *Staphylococcus aureus*: RN4220, SA081 and SA082. In addition, hourly impedance measurements were performed on four additional control test vials containing only MHB in order to track any conditioning effect of the impedance measurements on electrodes in the broth.

## 3. Results

### 3.1. RN4220 Growth

At the end of the experiments (17 h) the RN4220 vials containing sensors had reached cell densities of approximately 1 × 10^9^ CFU∙mL^−1^, which matched the RN4220 control vials. This indicates that, from an inoculation density of 1 × 10^6^ CFU∙mL^−1^, the sensors probably had no effect on bacterial growth. The normalised magnitude, phase angle, resistance and reactance readings, as discussed in Section 1, were examined for differences between the RN4220 cultures and the control cultures without bacteria. 

**Figure 2:**
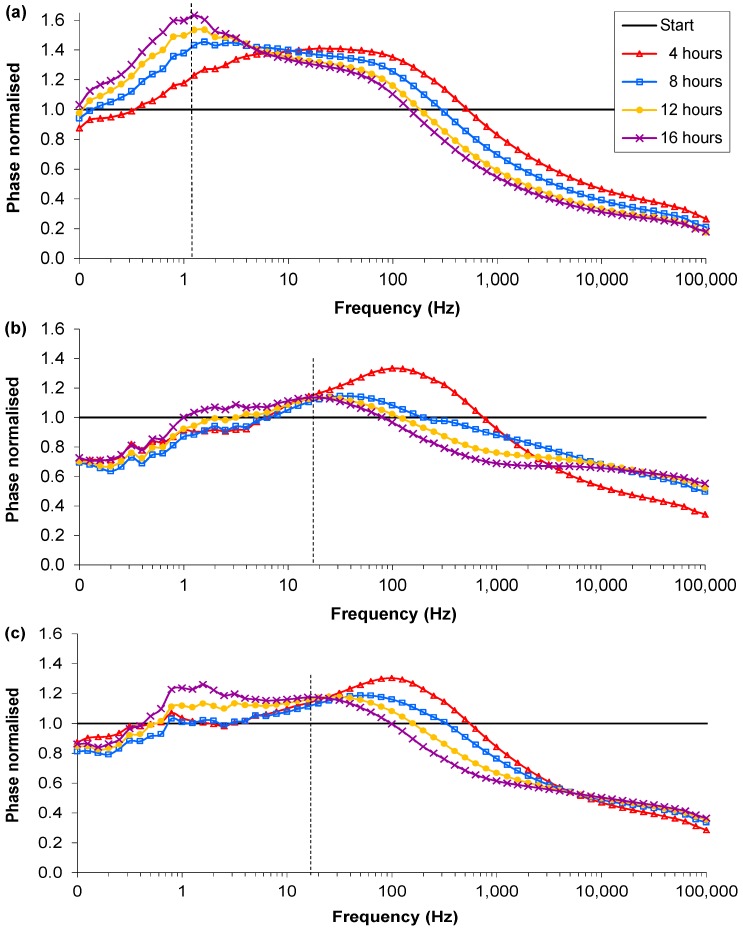
Examples of the normalised phase angle profiles from the single suspension experiments: (**a**) Control, MHB-only; (**b**) RN4220 culture 1; (**c**) RN4220 culture 2. Dotted line indicates the normalized phase angle peak.

The normalised profiles showed some clear trends and differences between the control vial and the vial with bacterial suspension. The most significant difference in the normalised magnitude was that in the MHB-only vials the value of the reading between 0.1 and 1 Hz decreased to a value of 0.5 while the RN4220 culture vials decreased to values of 0.4 and 0.3. However the discernible differences were more pronounced in the normalised phase angle. In the control (MHB-only), a peak in the normalised phase angle occurred between 50 and 100 Hz at the start and decreased over time to between 0.79 and 2.00 Hz at 17 h (Figure 2(a)). The normalised phase angle peak of the control (MHB-only) descended below 10 Hz between 4 and 10 h across all five separate samples. In contrast, in the RN4220 cultures, the normalised phase peak after 10 h remained between 32 and 20 Hz and hence never descended below 10 Hz (Figure 2(b)). This was repeatable over 5 separate culture experiments. Therefore it appears that the two cultures do have separate signature traces in the phase angle over frequency and is the first indication that peaks in signature traces of phase angles in normalised impedance plots could distinguish between the absence and/or growth of bacteria. In three of the five RN4220 cultures, a secondary peak appeared at a frequency less than 10 Hz and reached a higher magnitude by 17 h than the primary peak (Figure 2(c)). However the primary peak was still visible and allows the RN4220 cultures to be distinguished from the MHB-only samples by comparison of the normalised phase angle plots. 

**Figure 3:**
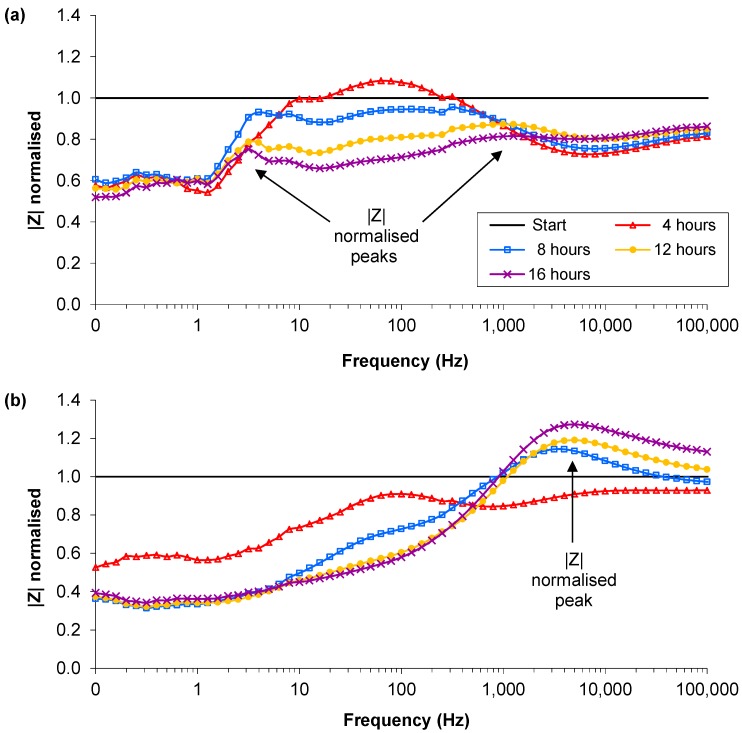
Examples of the normalised magnitude profiles from the single suspension experiments: (**a**) Control, MHB and 0.2% glucose; (**b**) RN4220 in MHB and 0.2% glucose. Arrows indicate peaks of interest.

There were similar discernible differences in the normalised phase peak between the MHB with glucose and the RN4220 culture in MHB with glucose. The normalised phase peak at 17 h was between 1 and 2 Hz in all five media-only cultures, and between 10 and 32 Hz for the RN4220-glucose cultures. In addition to the differences in the normalised phase peaks the normalised magnitude peaks showed clear differences with the added glucose. In the MHB-glucose-only vials by 17 h a first peak had occurred between 1 and 3 Hz and a second one between 200 and 1.3 kHz (Figure 3(a)). In contrast, in the RN4220-glucose experiments the first and second peaks were not present at the end of the experiment, however a third peak between 2.5 k and 6.3 kHz was present, as shown in Figure 3(b). The change in the peaks shown in the RN4220 cultures occurred between 4 and 8 h after inoculation. This was repeatable over five separate culture experiments and is the first evidence that discernible differences in the normalised phase angle profiles occur due to the presence of bacteria, depending on the media contents.

### 3.2. Lower Inoculation Densities, Glucose and Pre-washing

**Figure 4:**
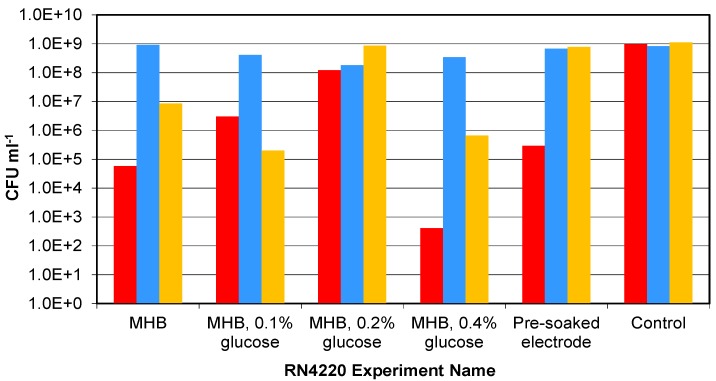
The final cell numbers of RN4220 in the parallel suspension experiment from low starting cell densities. Bar colours indicate sample number: Red—sample one; Blue—sample two; and Yellow—sample three.

The final cell densities of RN4220 after 16 h culture with Ag-AgCl sensors in MHB, MHB with glucose and with pre-washed sensors, showed wide variation, as shown in Figure 4. The control cultures, which contained no sensors, always reached approximately 1 × 10^9^ CFU∙mL^−1^ within 24 h. Only one of the three vials with sensors in MHB grew to a density in excess of 5 × 10^7^ CFU∙mL^−1^. In the experiments with added glucose and sensors present, one MHB-0.1% glucose, three MHB-0.2% glucose and one MHB-0.4% glucose vials grew to more than 5 × 10^7^ CFU∙mL^−1^. For the sensors that were pre-washed before inoculation, only two of the three vials reached densities above 5 × 10^7^ CFU∙mL^−1^. In total there were 15 RN4220 vials with sensors, and of these 15 it was observed that 8 reached cell densities above 5 × 10^7^ CFU∙mL^−1^, and the remaining 7 remained below this level. This was evidence that the presence of the Ag-AgCl sensors may have inhibited the RN4220 growth from lower inoculation densities. The level of glucose appears to affect the amount of inhibition, with 0.2% being the optimum to overcome the reduced growth.

**Figure 5:**
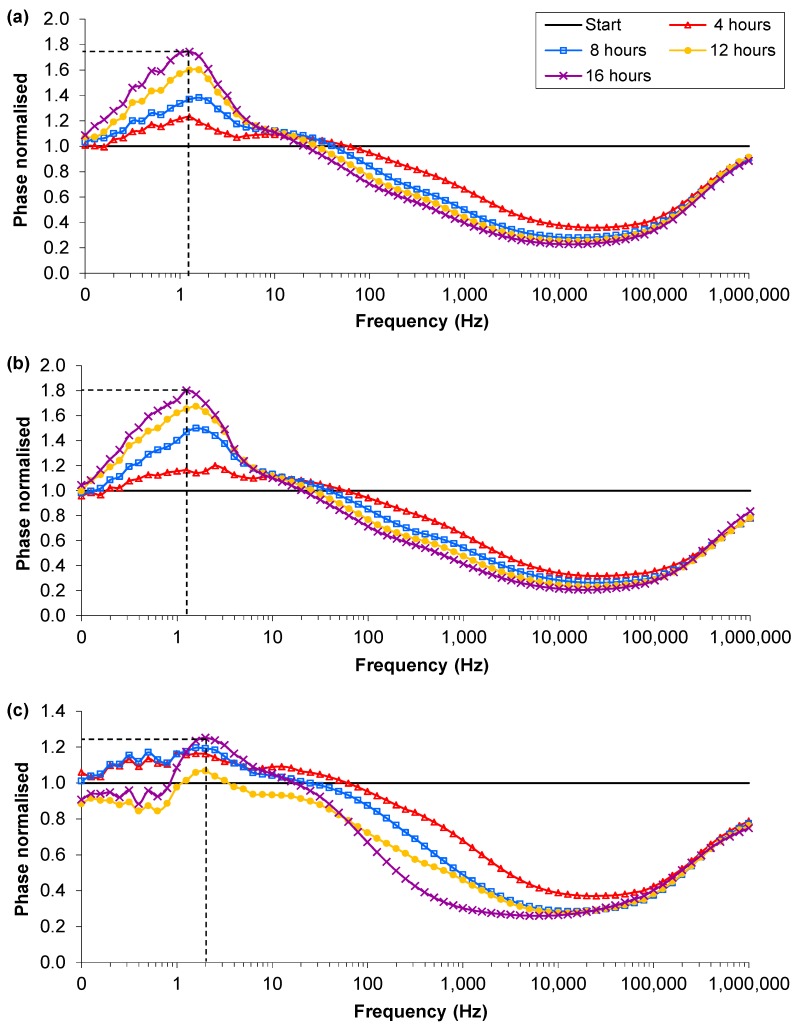
An example of the normalised profiles with Ag-AgCl sensors in parallel suspensions: (**a**) MHB; (**b**) MHB and low density RN4220 at 16 h; (**c**) MHB and high density RN4220 at 16 h. Dotted lines indicates the normalised phase angle peak at 16 h.

The normalised phase angle profiles of the lower inoculation density experiments again showed the clearest differences between bacterial cultures and MHB–only vials with sensors present in both to record impedance. In all 24 vials, irrespective of the media type or preparation of the sensor, there were three types of normalised phase angle profiles. The profile for the controls, the MHB-only, contained a peak that increased in magnitude over time and at 16 hours was between 0.79 and 1.58 Hz (Figure 5(a)). These peaks were caused by a reduction in the resistance and capacitance from the background reading. There were two contrasting normalised phase angle profiles for the vials inoculated with low density RN4220 depending on the final cell density reached. The profiles of the low final cell density vials (<1 × 10^7^ CFU∙mL^−1^) were very similar to the media-only profiles, with magnitudes of the peak above 1.26 and frequencies between 0.79 and 1.58 Hz, and the difference between them could not be distinguished (Figure 5(b)). These profiles occurred in all of the inoculated vials and the final cell numbers were found to be less than 5 × 10^7^ CFU∙mL^−1^. The second RN4220 phase profile (Figure 5(c)) occurred in vials inoculated with low starting density RN4220 but in these the final cell densities were between 1 × 10^8^ and 1 × 10^9^ CFU∙mL^−1^. The magnitudes of the peak were below 1.26 and the frequencies of the peaks between 1.58 and 2.00 Hz. This change in the peak was caused by a larger decrease in the resistance and an increase in the capacitance, potentially indicating the increase in metabolites and cell membranes respectively. Therefore it appears that once bacterial cultures reach a sufficient cell density a separate signature trace in the normalised phase angle over frequency occurs and indicates that comparing signature traces could discern between the absence and or/growth of bacteria above certain densities.

**Figure 6:**
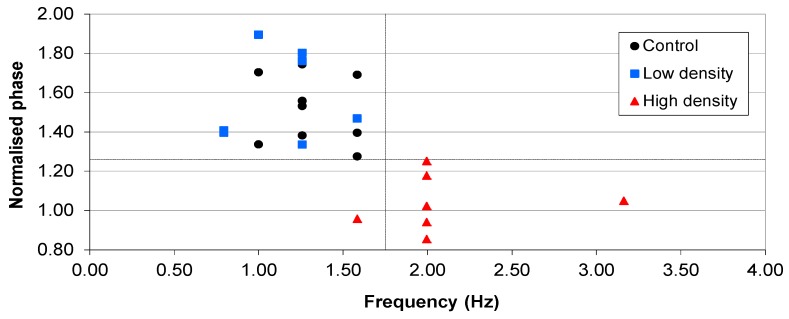
The magnitude and frequency of the normalised phase angle peak from all 24 vials from the parallel suspension experiments. The majority of the vials with cell densities that reached above 1 × 10^7^ CFU∙mL^−1^ occurred in the lower right quadrant, while the media only and lower density vials occurred in the upper left quadrant.

Examination of the magnitude and frequency of the normalised phase angle peak at 16 h shows a distinction in magnitude between the RN4220 cultures that reached above 5 × 10^7^ CFU∙mL^−1^ and those that did not. However the frequency of the peak is not completely distinct due to overlapping frequency ranges at approximately 1.58 Hz (Figure 6). This has obvious implications for a detection system that uses frequency bands to discern the absence or presence of bacteria.

### 3.3. Presence of Ag-AgCl Sensors

**Figure 7:**
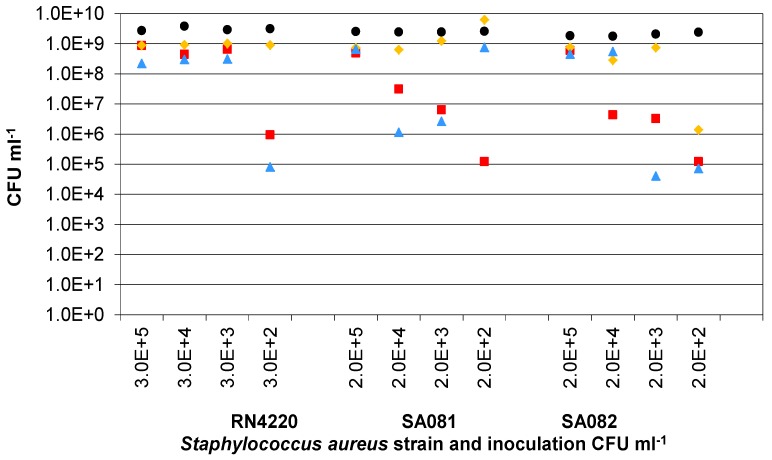
The final numbers of RN4220, SA081 and SA082 with Ag-AgCl sensors after 24 h culturing from different starting densities. Three replicate cultures are shown for each of the three strains of *Staphylococcus aureus* at each starting density. Sample 1(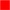); Sample 2(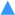); Sample 3(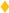); Mean control (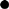).

To investigate the effect of the inoculation density on the bacterial growth in the presence of Ag-AgCl sensors, the presence of which will introduce some silver ions into the culture system, the three strains of *Staphylococcus aureus* were grown from four starting cell densities between 1 × 10^2^ and 1 × 10^5^ CFU∙mL^−1^ without the application of voltage for impedance measurements (Figure 7). This would also show whether the clinical strains were more robust in the presence of the AgCl than RN4220. The final cell densities with sensors indicated that, below a critical starting cell density, it was not guaranteed that the final cell densities would reach 1 × 10^9^ CFU∙mL^−1^ (expected level based on the RN4220 controls without sensors). For RN4220, this critical starting density was approximately 1,000 CFU∙mL^−1^ while, for SA081 and SA082, the density was approximately 1 × 10^5^ CFU∙mL^−1^. This provides further evidence that the presence of the Ag-AgCl sensors has an influence on bacterial growth rates and that the two clinical strains may not be as resistant to AgCl as the RN4220 strain.

### 3.4. Influence of Impedance Measurement

**Figure 8:**
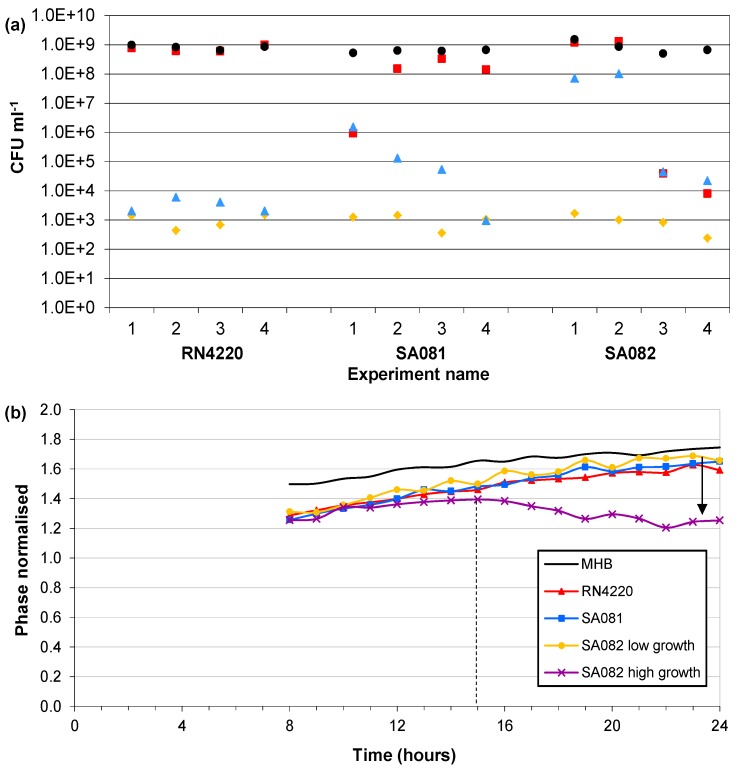
(**a**) The final cell numbers of RN4220, SA081 and SA082 with Ag-AgCl sensors after 24 h with and without the application of impedance measurements. Four replicate cultures are shown for each of the three strains of *Staphylococcus aureus*. (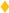) Start value; (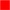) Final cell density without impedance measurements; (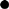) Control growth with no sensors; (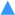) Final cell density with impedance measurements. (**b**) Typical magnitudes of the normalised phase angle peak over time from the influence of impedance measurement experiments. The peak occurs at approximately 8 h and increases over time unless the bacteria reach cell densities in excess of 1×10^7^ CFU∙mL^−1^ where upon it starts to decrease.

The final experiment investigated the influence of applying voltage to the electrodes, with impedance measurements and Ag-AgCl sensors, on the bacterial growth of the three *Staphylococcus aureus* strains. The controls of all three strains, which contained no sensors, always reached approximately 1 × 10^9^ CFU∙mL^−1^ within 24 h. The strain RN4220 reached control levels without impedance measurements. However the 4 separate vials with impedance measurements had final cell densities between 2 × 10^3^ and 6 × 10^3^ CFU∙mL^−1^ (Figure 8(a)). In the vials without impedance measurements, the strain SA081 reached 1 × 10^8^ CFU∙mL^−1^ in three vials and only 1 × 10^6^ CFU∙mL^−1^ in the fourth. With impedance measurements the SA081 strain reached cell densities between 1 × 10^3^ and 1 × 10^6^ CFU∙mL^−1^. The strain SA082 without impedance measurements reached control densities in two vials and only 1 × 10^4^ CFU∙mL^−1 ^in the other two. Similarly, the strain SA082 had two vials reaching 1 × 10^8^ CFU∙mL^−1^ and two vials reaching 3 × 10^4^ CFU∙mL^−1^ with impedance measurements. These results suggest that performing impedance measurements every hour may have an influence on the bacterial growth. These final cell densities clearly show that the presence of the Ag-AgCl electrodes inhibits normal growth rates and that the application of electrical voltage during the impedance measurements increases this reduction.

The normalised phase angle profiles from the influence of impedance measurement experiments were expected to be similar to those detailed in Section 3.2. The frequency of the normalised phase angle peak occurred in all 4 control and 12 bacterial cultures between 0.8 and 2 Hz with no discernible differences between the controls and bacterial cultures. However the differences in magnitude changes of the peaks as seen in the parallel suspension experiments were observed (Figure 8(b)). The normalised phase angle peaks of the 4 media-only and 10 low density vials of RN4220, SA081 and SA082 started at approximately 8 h and increased over time to magnitudes above a value of 1.5. In contrast the peaks of the two high density SA082 vials increased until 13 to 14 h and then decreased for the remaining hours. Despite the low number of samples displaying high cell densities it appears that the examination of normalised phase profiles of multiple strains of *Staphylococcus aureus* shows a difference in the signature trace when growth is in excess of 5 × 10^7^ CFU∙mL^−1^ compared with low density cultures (<1 × 10^6^ CFU∙mL^−1^) or media only. This is the first evidence that the phase signature traces over frequency can be used to discern high growth levels in multiple strains of *Staphylococcus aureus.*

## 4. Discussion

### 4.1. Detection of Bacteria

The normalised impedance profiles over frequency have shown a novel way of detecting the presence and/or absence of bacteria in real time. The analysis of the profiles for unique signature traces will indicate the presence of *Staphylococcus aureus* strains in excess of 5 × 10^7^ CFU∙mL^−1^, which is in accordance with the detection limits of earlier studies of bacteria growth with impedance [8]. These earlier studies on bacteria in bulk fluids, Ur and Brown [11], Cady [6], Richards *et al.* [10], and Firstenberg-Eden and Eden [7], for example, concentrated on specific frequencies and produced characteristics curves over time for different species of bacteria. Firstenberg-Eden and Eden [7] also produced a mathematical model for calculating the starting cell density. The limitations of these systems were that they required controlled conditions to reproduce the curves and parameters known about the bacteria to calculate the cell densities. The advantage of the normalised impedance profiles over frequency is that once the signature traces are known the controls are not required. This may be ideal for monitoring wounds for infection in real time when a control would not be possible.

The mechanisms that have the potential to cause differences in the normalised impedance of biological systems within the frequency range examined are counterion diffusion effects and capacitive charging of cell membranes. At low frequencies (between 10 Hz and a few kilohertz) the impedance is characterised by counterion diffusion effects while the capacitive charging of cell membranes occurs in the tens of kilohertz to tenths of a megahertz range [5]. The counterion diffusion effect is caused by the cell membranes acting as large capacitors and the current flowing only through the extracellular media. The impedance within the capacitive membrane charging frequency range is governed by cell membrane reactance. As the frequency increases the membrane is not fully charged therefore decreasing the reactance. In addition this allows more current to flow inside the cell and hence the resistance decreases.

The relative changes over time observed in the normalised phase profiles with MHB-only may be due to oxidation of the electrode surface or absorption of molecules onto the electrode surfaces. In the RN4220 cultures, the presence of the cells produced further changes observed in the normalised phase profiles. One explanation for these additional changes is that they are due to the metabolism of nutrient molecules to smaller and more conductive ones. An increase in conductivity of bacterial cultures was previously explained by changes to smaller and more highly charged molecules in the media [7]. Another possibility is that this may be linked to the increased number of suspended cells and their membranes. Finally, although there may be some bacterial attachment to the electrodes the major influence on the impedance in this experimental arrangement is the growth of the organisms in the bulk broth. Of course, as the technique is transferred to wound dressings then attachment to the electrodes may become a much more important influence. 

The contents of the media can influence the impedance profiles, as observed in the single suspension experiments when 0.2% glucose was added to the MHB. In comparison to the experiments without glucose, there were noticeable differences in the normalised impedance magnitude profiles between the controls and the bacterial cultures. The profile for the RN4220 cultures with 0.2% glucose showed an additional magnitude peak between 2.5 k and 6.3 kHz. This additional peak may have been caused by a change in the structure of the cell walls since it has been shown that the available nutrients, including carbon have an influence on the structure and composition of *Staphylococcus aureus* cell walls [26]. Another possibility is an additional metabolic product is produced when glucose is present, for example, bacteria metabolise glucose to lactic acid and then to carbonic acid [7]. This would alter the media composition and the interaction between the cells and media could have caused an additional peak in the normalised magnitude. 

There were an increased number of cell replications from the lower starting cell density in the parallel experiments. The glucose would have been used for energy and basic cell construction rather than modified cell structures. This may be the reason why the additional magnitude peak was not observed in the parallel experiments with glucose. In the intended application of monitoring wounds, the availability of nutrients may be restricted in the host [27] and therefore the signature traces as a result of the bacteria-glucose interaction may also not occur. 

Another consideration is whether in an actual wound detecting 5 × 10^7^ CFU∙mL^−1^ would be adequate or actually be a late warning system given that infections are more likely to occur at cell densities greater than 1 × 10^5^ CFU per gram of tissue [3]. Correlations between swab cultures and tissue biopsies have been shown, for example, 1 × 10^5^ CFU per gram of tissue is equivalent to approximately 5.9 × 10^5^ CFU per swab of a square centimetre [28]. However, it is still unclear how bacteria levels per tissue gram or per cm^2^ would relate to a detection system that indicates 5 × 10^7^ CFU mL^−1^. The levels of bacteria in a wound must be evaluated with other important factors, including bacterial virulence and host resistance [29]. Therefore valuable information given by an infection monitoring system for wounds could be used to indicate, like semi-quantitative swabs, the growth between light (1+) and profuse (4+) cultures [3]. This would provide information to the healthcare personnel that could be used in addition to other factors to select the most appropriate treatment options.

### 4.2. Bacterial Growth

The bacterial growth experiments from different densities suggest that below a critical cell density the Ag-AgCl sensors are slowing or inhibiting growth. The probable cause is silver ions, which are known to inhibit bacterial growth by cell membrane damage and damage to associated proteins [30,31]. Alternatively silver salts have been shown to enhance or diminish the mechanism of silver inhibition depending on the concentration of the anions [32,33]. Therefore there may have been differences in precipitation of AgCl between individual samples.

The range of cell densities observed in the experiments described in Section 3.2 may have been affected by the media contents, sensor preparation and presence of impedance measurements. In all three experimental runs the RN4220 culture with 0.2% glucose added to the MHB reached the levels of control which could be related to a glucose concentration for an optimum growth rate. The pre-washed sensors reached the control levels in two of the three experimental runs possibly indicating that pre-washing of the sensors is a solution to the problem of inhibition. The one pre-washed sensor vial that did not achieve the control levels of growth may have been poorly washed. 

The possibility of the impedance measurements having a detrimental effect on the bacterial growth was confirmed in the influence of impedance measurement experiments on all three *Staphylococcus aureus* strains. This possibly indicates that the measurements were causing more silver to be released into the media or that the electric current was damaging the culture. This is surprising since the total current density for the short time of measurement was in the range of 50 A∙m^−2^ at a voltage of 200 mV rms. Inhibition of bacteria by electrical stimulation generally uses constant currents or high voltage pulsed currents for prolonged periods, hours or days, for example [34]. Also an alternating current did not inhibit *Escherichia coli* compared with a direct current [35]. Therefore it would seem unlikely that an alternating current that is applied for approximately 2 min every hour directly causes the inhibition.

It is worth mentioning that a chronic wound *in vivo* is a complex environment with many additional factors potentially effecting the normalised impedance measurements, for example the dressing material, moisture levels, and the differences between colonisation, active infection and biofilm growth. Dressing materials that release silver or iodine will affect the measurements in a similar manner to the silver being released from the Ag-AgCl electrodes and therefore the bacteria are likely to still produce a noticeable change in the normalised impedance. Moisture levels are unlikely to be an issue with an active infection because infected wounds tend to produce more exudate than colonized wounds. The effect of biofilms on normalised impedance profiles has been investigated in a companion study.

## 5. Conclusion

This paper has shown that, by using electrical impedance with screen-printed disposable Ag-AgCl sensors in bacterial cultures and analysing the normalised impedance profiles over the frequency spectrum, the presence and/or absence of bacteria can be monitored in real time. Clearly the results obtained relate only to *Staphylococcus aureus* and work is now required to look at other types of bacteria and multiple organism cultures to check for the relevant impedance signatures. If signatures for other types of bacteria can be clearly identified then this novel system would be ideal to monitor infection underneath dressings, allowing healthcare personnel to make more informed decisions about changing dressings and administrating the best treatment. Recently, the presence of biofilms in wounds has been highlighted as one of the causes of delayed wound healing. Therefore, examining the impedance signature traces over frequency with biofilm growth on the disposable electrodes will also be a part of future work. The concept described here is the subject of a patent application by the Univerity of Strathclyde [16].
